# Gastric bezoar entering the duodenum causing intestinal obstruction: A case report

**DOI:** 10.1097/MD.0000000000044149

**Published:** 2025-09-05

**Authors:** Haixia Wang, Yanliang Zhang, Huang Zhong, Hongbin Zhang, Yong Ma

**Affiliations:** a Department of Gastroenterology, ZiGong First People’s Hospital, Zigong, Sichuan Province, China; b Department of General Surgery, ZiGong First People’s Hospital, Zigong, Sichuan Province, China.

**Keywords:** bezoar, bowel obstruction

## Abstract

**Rationale:**

Gastric bezoar-induced duodenal obstruction represents a rare clinical entity with <0.5% incidence among mechanical bowel obstructions.

**Patient concerns:**

A 73-year-old female patient presented to our institution, manifesting acute abdominal syndrome characterized by progressive pain and distension.

**Diagnoses:**

Computed tomography demonstrated a 3.5 cm intraluminal phytobezoar at the duodenal horizontal segment and proximal small bowel dilatation; Intraoperative: Bezoar impaction 60 cm from the ileocecal valve.

**Interventions:**

Initial conservative therapy included: fasting, anti-infection, acid suppression, indwelling gastric tube. After 72 hours of failed conservative management, laparoscopic-assisted jejunotomy was performed.

**Outcomes:**

Resolution of obstructive symptoms within 6 hours post-op and return of bowel function at 48 hours.

**Lessons:**

This diagnostically challenging case underscores the critical importance of obtaining detailed dietary histories (particularly persimmon/fig consumption) in the evaluation of mechanical bowel obstruction.

## 1. Introduction

Gastric bezoars are concretions formed within the gastrointestinal tract, predominantly retained in the gastric cavity. Through sustained peristaltic propulsion, these aggregates may migrate distally, potentially inducing mechanical small bowel obstruction with complications including strangulation, ulceration, and perforation. Definitive diagnosis is typically confirmed intraoperatively.^[1]^ Given the rarity of this condition coupled with its nonspecific clinical presentation, diagnostic challenges such as misdiagnosis or delayed recognition frequently occur. Early-stage bezoars may respond to conservative management including pharmacological dissolution or endoscopic retrieval. However, complex cases characterized by large dimensions (>5 cm) or migration beyond the pylorus often necessitate prompt surgical intervention.

Early clinical intervention through timely diagnosis and therapeutic management is crucial to mitigating complication risks in cases of gastrointestinal bezoars. While these concretions represent uncommon clinical occurrences, the migration of gastric bezoars into the jejunal lumen with subsequent mechanical obstruction constitutes an exceptionally rare phenomenon within surgical pathology. Notably, our institution recently managed a diagnostically challenging case of jejunal obstruction secondary to gastrogenic bezoar migration, which we herein describe with comprehensive clinical correlation.

## 2. Case report

A 73-year-old female patient presented to our institution on December 10, 2024, manifesting acute abdominal syndrome characterized by progressive pain and distension. The clinical course originated 24 hours before admission with acute-onset severe epigastric pain of undetermined etiology. The colicky pain pattern demonstrated paroxysmal intensification without radiation or identifiable exacerbating/relieving factors. Concomitant symptoms included intractable nausea, bilious emesis (6 episodes), complete cessation of flatus and bowel movements, and progressive abdominal tympanites. Notably absent were febrile response, diarrheal symptoms, or hematochezia.

**Figure 1.**
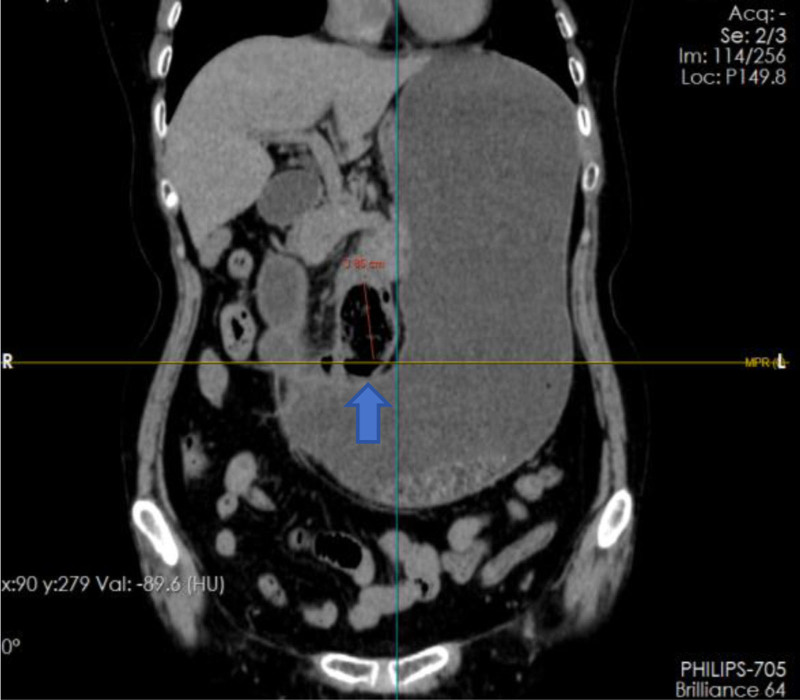
Location of the stone on abdominal CT (coronal view).

**Figure 2.**
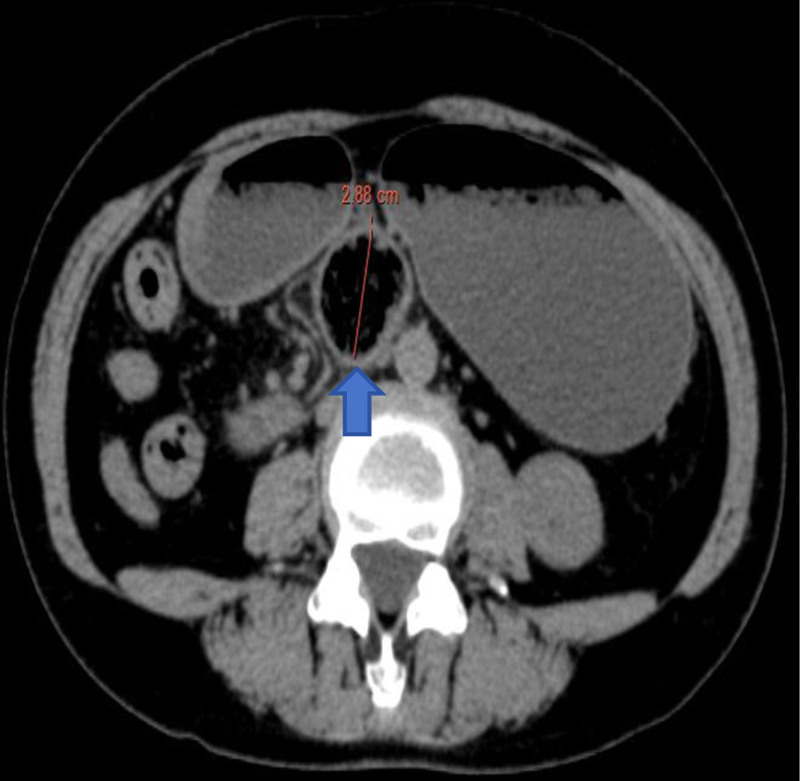
Location of the stone on abdominal CT (axial position).

Physical examination revealed abdominal tenderness localized to the epigastrium without rebound phenomena or palpable masses. Negative Murphy sign and diminished bowel sounds (3/min) were documented. Emergency contrast-enhanced abdominal computed tomography (CT) demonstrated a 3.5 cm intraluminal phytobezoar at the duodenal horizontal segment, accompanied by mural thickening (4.8 mm) and proximal luminal dilation (duodenal diameter 4.2 cm, gastric antrum 8.1 cm) consistent with mechanical obstruction (Figs. 1 and 2). Laboratory analysis revealed leukocytosis (white blood cell count 11.18 × 10⁹/L, neutrophils 88.8%, absolute count 9.93 × 10⁹/L; lymphocytes 8.9%, absolute count 1.0 × 10⁹/L) and elevated hypersensitive C-reactive protein (5.22 mg/L).

### 2.1. Final diagnosis

(1) Mechanical intestinal obstruction secondary to jejunal phytobezoar impaction.
(2) Migratory gastrogenic bezoar (duodenal-to-jejunal transition).

### 2.2. Therapeutic course

Initial conservative therapy included: fasting, anti-infection, acid suppression, indwelling gastric tube.

### 2.3. Imaging evolution

**Figure 3.**
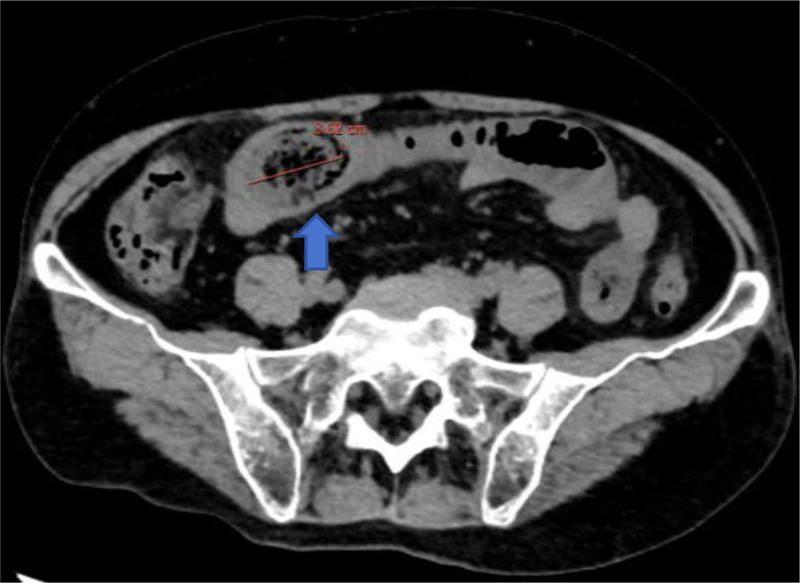
Maximum diameter of stones.

(1) Repeat contrast-enhanced CT (12/13/2024) (Fig. 3) (vs duodenal position on 12/10/2024) demonstrated: 3.5 cm phytobezoar migration 45 cm distal to the ligament of TreitzProgressive mural edema (wall thickness 6.2 vs 4.8 mm initial).
(2) Transition zone dilatation (jejunal diameter 5.1 vs 4.2 cm previously).
(3) New “double halo” sign in the adjacent mesentery.

### 2.4. Surgical intervention

**Figure 4.**
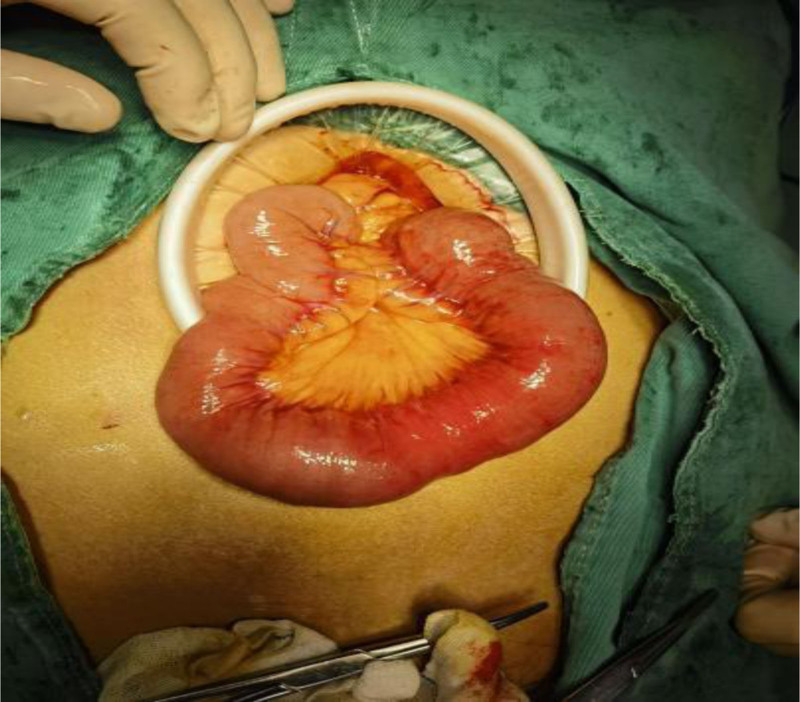
Location of the stone (at surgery).

**Figure 5.**
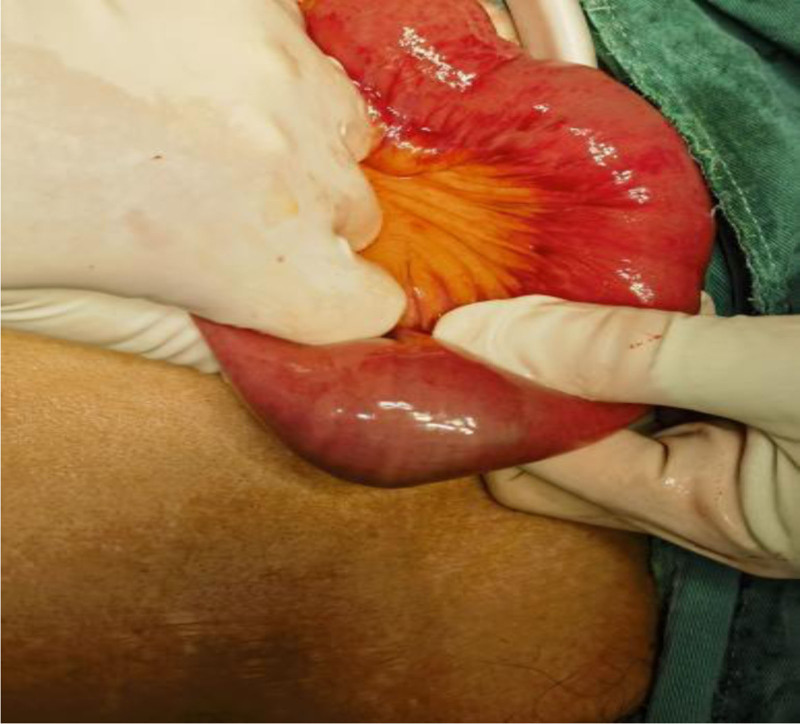
Location and shape of the stone.

**Figure 6.**
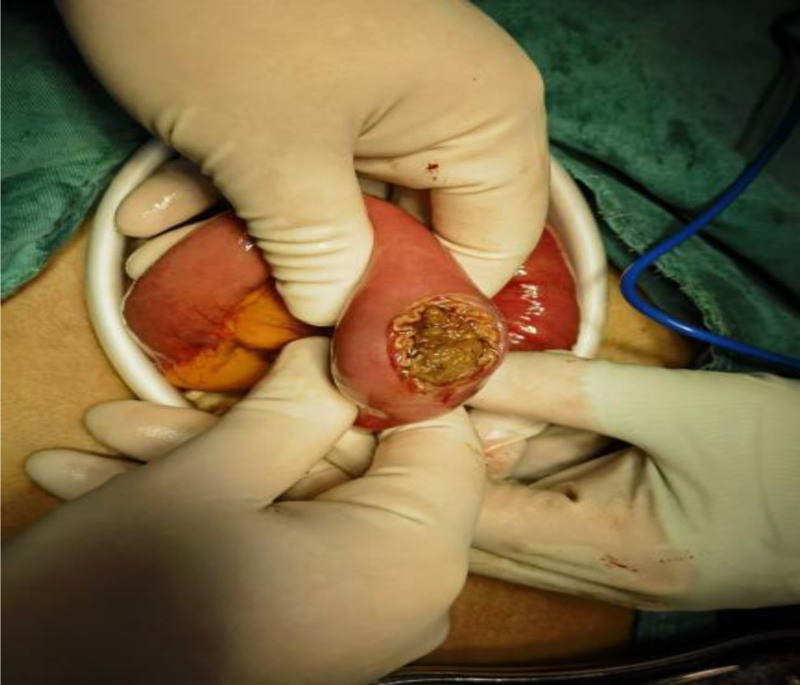
Cut open the duodenum to remove the stone.

**Figure 7.**
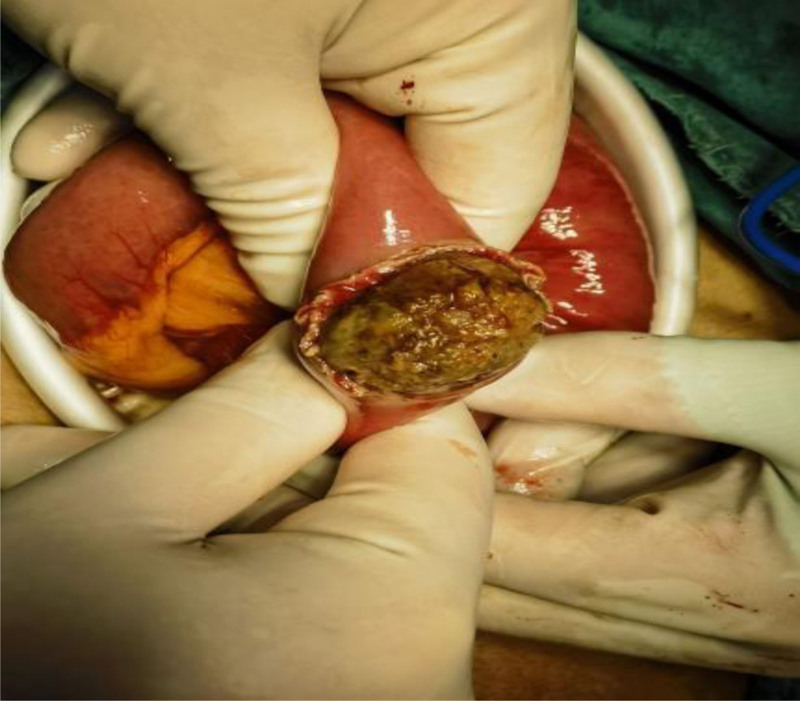
Extruded the stone.

**Figure 8.**
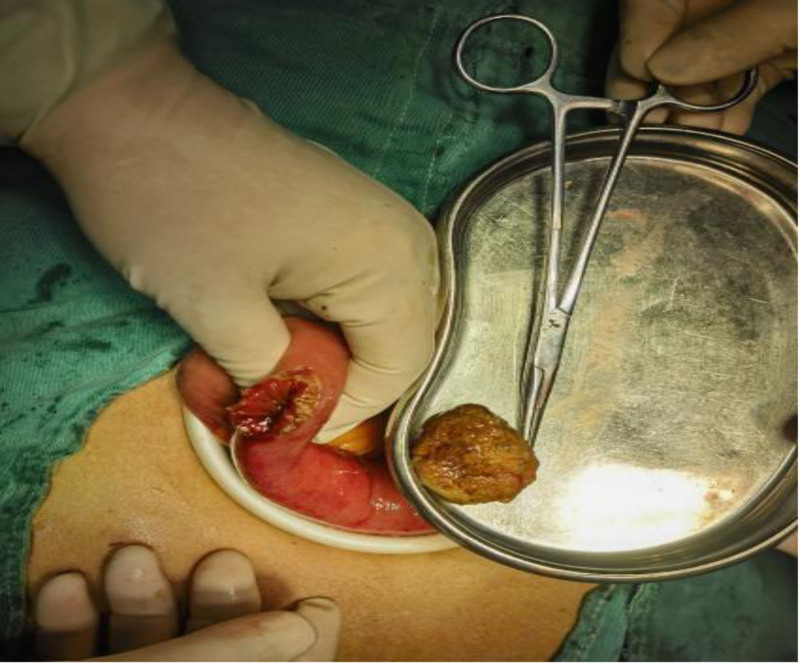
Stone removal.

**Figure 9.**
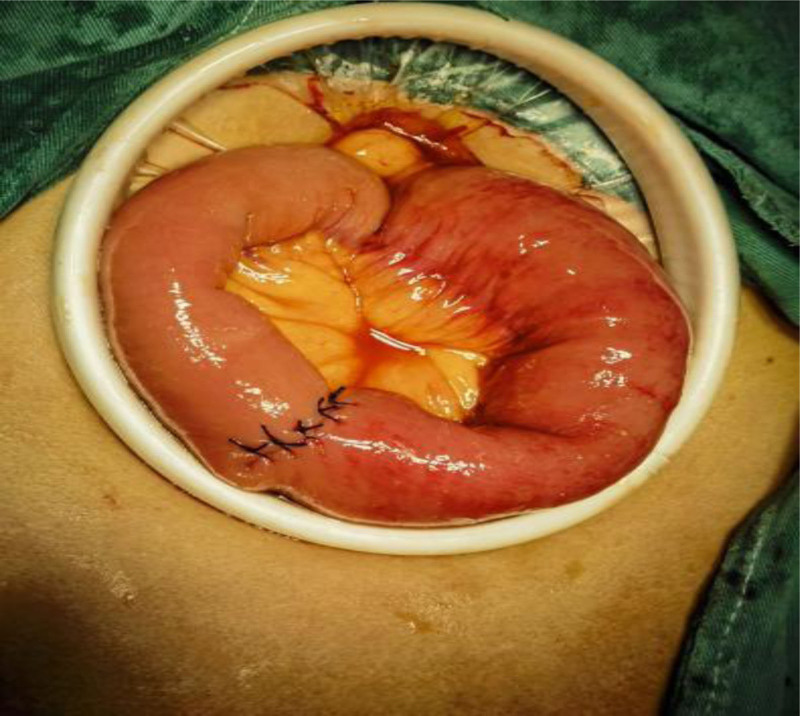
Suture the wound.

After 72 hours of failed conservative management, laparoscopic-assisted jejunotomy was performed (Figs. 4–9):

(1) Intraoperative findings: Bezoar impaction 60 cm from the ileocecal valve.
(2) Specimen characteristics: 4.0 × 3.5 × 4.0 cm irregular mass (gross weight 32 g).
(3) Histopathology: Laminated plant cellulose matrix with 95% tannin concentration (Folin-Ciocalteu assay).

### 2.5. Postoperative outcomes

(1) Resolution of obstructive symptoms within 6 hours postoperatively.
(2) Return of bowel function at 48 hours (first flatus documented).

Discharge criteria met on postoperative day 5:

(1) Tolerating a regular diet.
(2) White blood cell count normalized (8.7 × 10⁹/L).
(3) No surgical site complications (Clavien-Dindo grade 0).

## 3. Discussion

Small bowel obstruction typically results from mechanical luminal compromise, with common etiologies encompassing phytobezoars, ascaris boluses, and fibrous food impactions. Phytobezoar-induced small bowel obstruction represents a rare clinical entity, accounting for 0.4% to 4.8% of all intestinal obstructions.^[2]^ Population-based studies estimate the general prevalence of gastrointestinal bezoars at <1%, with gastric localization observed in <0.5% of esophagogastroduodenoscopy procedures.^[3]^ This epidemiological profile underscores their frequent underrecognition in clinical practice.

Gastric phytobezoars form through the polymerization of tannin-rich substances (e.g., persimmon diospyrobezoars) with gastric acid-derived mucopolysaccharides, creating insoluble concretions. While 85% to 90% of gastric bezoars undergo spontaneous fragmentation in alkaline intestinal environments, persistent concretions >2.5 cm may migrate distally, acting as a nidus for progressive size accretion through deposition of indigestible fibers—a process exacerbated by intestinal dysmotility.^[4]^ Jejunal impaction typically occurs at anatomical transition zones (e.g., Treitz ligament), manifesting as “rolling obstruction” characterized by intermittent symptom relief followed by acute exacerbations.

Multidetector CT emerges as the gold standard for bezoar identification, achieving 88% to 94% sensitivity through pathognomonic findings: Intraluminal mottled gas pattern (“bubbly sign”): Focal bowel wall thickening with proximal dilatation and Absence of oral contrast progression.

Endoscopic evaluation remains indispensable for gastric bezoar characterization and therapeutic intervention, particularly for diospyrobezoars exhibiting endoscopic refractoriness to chemical dissolution.^[5]^

In clinical treatment decision-making, patient management is categorized into conservative therapy and invasive intervention based on disease severity.

### 3.1. Indication comparison

Conservative therapy is primarily indicated for patients with bezoars smaller than 2 cm without ischemic manifestations or those presenting with partial bowel obstruction. Invasive intervention is warranted in severe cases, including failure of conservative treatment, complete bowel obstruction, and the presence of peritoneal signs (e.g., tenderness, rebound pain, and rigidity).

### 3.2. Therapeutic modalities

The conservative approach comprises 3 key measures:

(1) Fasting combined with prokinetic agents to reduce gastrointestinal burden and enhance motility.
(2) Coca-Cola lavage, utilizing its acidic properties to dissolve bezoars.
(3) Cellulase or papain enzyme instillation to enzymatically degrade bezoars.

Invasive interventions involve 3 escalating surgical options:

(1) Endoscopic fragmentation as the first-line minimally invasive procedure.
(2) Laparoscopic enterotomy for stone extraction when endoscopic access is limited.
(3) Resection-anastomosis of necrotic bowel segments as a life-saving measure in cases of intestinal wall necrosis.

This tiered treatment framework prioritizes severity-driven escalation from noninvasive to minimally invasive strategies, balancing therapeutic efficacy with patient safety.

### 3.3. Critical surgical principles

 (1) Intraoperative pan-enteric palpation to exclude synchronous bezoars (15% multiplicity rate).

(2) Primary enterotomy closure with interrupted 4 to 0 absorbable sutures.
(3) Post-decompression nasogastric drainage maintenance (48–72 hours).
(4) Postoperative cellulase supplementation (3–5 days).
3.4. Preventive framework

High-risk populations (gastroparesis/diabetic patients) should receive:

(1) Dietary counseling against tannin-rich foods.
(2) Quarterly prokinetic regimens.
(3) Annual surveillance endoscopy.

## 4. Conclusion

### 4.1. Clinical implications and preventive strategies

This diagnostically challenging case underscores the critical importance of obtaining detailed dietary histories (particularly persimmon/fig consumption) in the evaluation of mechanical bowel obstruction. Our experience reinforces the necessity of incorporating the “DISH” mnemonics into initial assessments:

(1) Dietary patterns (tannin-rich foods).
(2) Ingested foreign bodies history.
(3) Surgical history (prior gastrectomy/gastric bypass).
(4) Habits (mastication disorders).

## Acknowledgments

The authors would like to thank the patient, for his contribution in this research.

## Author contributions

**Conceptualization:** Haixia Wang, Yanliang Zhang.

**Data curation:** Haixia Wang, Yanliang Zhang.

**Formal analysis:** Haixia Wang, Yanliang Zhang.

**Investigation:** Hongbin Zhang.

**Methodology:** Haixia Wang, Hongbin Zhang.

**Project administration:** Hongbin Zhang.

**Resources:** Hongbin Zhang, Yong Ma.

**Software:** Yong Ma.

**Supervision:** Huang Zhong, Yong Ma.

**Validation:** Yong Ma.

**Visualization:** Yong Ma.

**Writing – original draft:** Haixia Wang, Yong Ma.

**Writing – review & editing:** Haixia Wang, Huang Zhong, Yong Ma.
